# MARCH8 Restricts Influenza A Virus Infectivity but Does Not Downregulate Viral Glycoprotein Expression at the Surface of Infected Cells

**DOI:** 10.1128/mBio.01484-21

**Published:** 2021-09-14

**Authors:** Fernando Villalón-Letelier, Andrew G. Brooks, Sarah L. Londrigan, Patrick C. Reading

**Affiliations:** a Department of Microbiology and Immunology, The University of Melbourne at the Peter Doherty Institute for Infection and Immunity, Victoria, Australia; b WHO Collaborating Centre for Reference and Research on Influenza, Victorian Infectious Diseases Reference Laboratory, The Peter Doherty Institute for Infection and Immunity, Victoria, Australia; Columbia University/HHMI

**Keywords:** RNA virus, glycoproteins, influenza, ubiquitination

## Abstract

Membrane-associated RING-CH8 (MARCH8) impairs the cell surface expression of envelope glycoproteins from different viruses, reducing their incorporation into virions. Using stable cell lines with inducible MARCH8 expression, we show that MARCH8 did not alter susceptibility to influenza A virus (IAV) infection, but virions released from infected cells were markedly less infectious. Knockdown of endogenous MARCH8 confirmed its effect on IAV infectivity. The expression of MARCH8 impaired the infectivity of both H3N2 and H1N1 strains and was dependent on its E3 ligase activity. Although virions released in the presence of MARCH8 expressed smaller amounts of viral hemagglutinin (HA) and neuraminidase (NA) proteins, there was no impact on levels of the viral HA, NA, or matrix 2 (M2) proteins detected on the surface of infected cells. Moreover, mutation of lysine residues in the cytoplasmic tails of HA, NA, and/or M2, or in the viral M1 protein, did not abrogate MARCH8-mediated restriction. While MARCH1 and -8 target similar immunological ligands and both restrict HIV-1, only MARCH8 inhibited IAV infectivity. Deletion of the N-terminal cytoplasmic (N-CT) domain of MARCH8 confirmed it to be a critical determinant of IAV inhibition. Of interest, deletion of the MARCH1 N-CT or its replacement with the MARCH8 N-CT resulted in acquisition of IAV restriction. Together, these data demonstrate that MARCH8 restricts a late stage in IAV replication by a mechanism distinct to its reported activity against other viruses. Moreover, we show that the N-CT of MARCH8 is essential for anti-IAV activity, whereas the MARCH1 N-CT inhibits its ability to restrict IAV.

## INTRODUCTION

Membrane-associated RING-CH (MARCH) family proteins are RING-finger E3 ubiquitin ligases consisting of 11 members that share a similar overall structure. MARCH family proteins generally comprise a N-terminal cytoplasmic tail containing a C4HC3 RING finger (RING-CH motif), two or more transmembrane (TM) domains, and a C-terminal CT domain, although MARCH7 and MARCH10 have no predicted TM domains (1, 2). These proteins were originally identified as cellular homologues of viral RING E3 ligases expressed by gammaherpesviruses and poxviruses, which promote immune evasion by downregulating the cell surface expression of major histocompatibility complex I (MHC-I) (3–5). It is now clear that mammalian MARCH proteins downregulate expression of a wide variety of cellular transmembrane proteins, including MHC-II, cluster of differentiation 86 (CD86), and transferrin receptor (TfR), among others (reviewed in reference 6). Some MARCH family members can be further classified into subgroups based on sequence conservation, which is particularly high (89%) across the RING-TM1-TM2 domains of MARCH1 and -8 (reviewed in reference 6). MARCH family subgroups share overlapping substrate specificity, with MARCH1 and -8 recognizing MHC-II, CD86, CD95, and TfR (reviewed in reference 6), although only MARCH8 was reported to downregulate CD44 and CD81 (7).

Recently, some mammalian MARCH-family proteins have also been identified to display antiviral activity through modulation of viral replication. Tada et al. first reported that human MARCH8 reduced the infectivity of HIV-1 and vesicular stomatitis virus glycoprotein (VSV-G)-pseudotyped viruses by downregulating envelope glycoproteins at the cell surface, resulting in reduced incorporation into nascent virions. Although this antiviral activity was E3 ligase dependent, subsequent studies demonstrated that MARCH8 targeted lysine (K) residues in the cytoplasmic tail (CT) of VSV-G, whereas HIV-1 Env downregulation occurred even if its entire CT was deleted (8–10). Similarly, CT deletion mutants of Ebola virus glycoprotein (EboV-GP) and severe acute respiratory syndrome coronavirus 2 (SARS-CoV-2) spike (S) glycoproteins remained sensitive to human MARCH8 (8). In addition, mouse MARCH1 and -8 were recently shown to target and downregulate the p15E envelope glycoprotein subunit of mouse leukemia virus (MLV) (9) and human MARCH1 and -2 both mediate antiviral activity against HIV-1 and VSV-G-pseudotyped viruses (9, 11). Together, these studies demonstrate that MARCH-mediated restriction is associated with downregulation of a broad range of viral envelope glycoproteins.

Herein, we demonstrate that MARCH8 expression was not associated with downregulation of viral hemagglutinin (HA), neuraminidase (NA), or matrix 2 (M2) protein from the surface of influenza A virus (IAV)-infected cells. Nevertheless, it mediated potent antiviral activity against IAV, since virions released from cells with doxycycline (DOX)-inducible MARCH8 expression showed reduced infectivity per particle and expressed smaller amounts of viral HA and NA proteins. Moreover, recombinant viruses with mutations in lysine residues in the cytoplasmic domains of HA, NA, and M2 or in the viral M1 protein (involved in virus budding from the plasma membrane [12]) all remained sensitive to MARCH8-mediated restriction, indicating that these viral proteins are not major targets for MARCH8-mediated ubiquitination. MARCH1 exhibits a high degree of sequence homology to MARCH8, targets similar immunological ligands, and mediates antiviral activity against HIV-1 (11). Although DOX-inducible MARCH1 and -8 both downregulated cell surface CD86, only MARCH8 mediated anti-IAV activity. Additional experiments confirmed the critical role of MARCH8 N-CT domain for anti-IAV activity and demonstrated that deletion of the MARCH1 N-CT, or its replacement with the MARCH8 N-CT, resulted in acquisition of IAV restriction. Together, these data indicate that MARCH8 mediates antiviral activity against IAV by a mechanism distinct from its reported activity against other viruses and identify the critical role of the N-CT domain for MARCH8-mediated restriction of IAV.

## RESULTS

### Generation of cell lines with inducible overexpression of MARCH1 and MARCH8 proteins.

**FIG 1:**
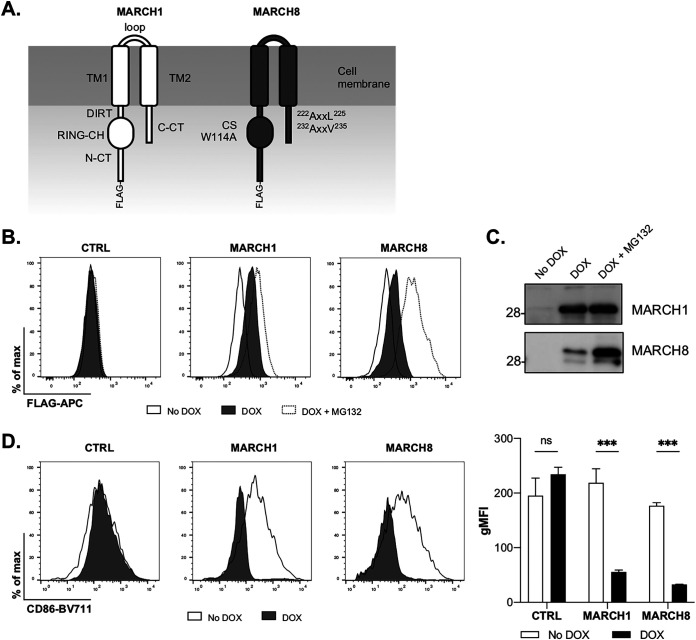
Inducible overexpression of functional MARCH1 and -8 proteins in 293T cells. (A) A schematic shows the domain organization of MARCH1 and MARCH8 proteins, including the N-terminal FLAG tag. (B) 293T cells were transduced with lentivirus to generate cell lines with stable DOX-inducible overexpression of FLAG-tagged MARCH1 and -8 proteins or untagged control (CTRL) protein. Cells were cultured for 24 h in media (No DOX) or supplemented with 1 μg/ml DOX (DOX) or with DOX and MG132 for the last 4 h (DOX+MG132). MARCH1 and -8 protein expression was assessed by intracellular staining with anti-FLAG antibody in conjunction with flow cytometry. Representative histograms are shown. (C) Cells in media (No DOX), DOX induced (DOX), or DOX induced in the presence of MG132 (DOX+MG132) were lysed, and proteins were subjected to immunoprecipitation, followed by SDS-PAGE under reducing conditions. Western blots were probed with anti-FLAG MAb to detect MARCH1 and -8. (D) Cells were transfected with a plasmid encoding CD86 and ZsGreen fluorescent protein. At 12 h post-transfection, MARCH1 and -8 expression was induced with DOX, as described above; 24 h later, the cells were stained for cell surface CD86. Representative histograms and the geometric mean fluorescence intensities (gMFI ± the SD) from triplicate samples are shown. Two-way analysis of variance (ANOVA), using Bonferroni’s posttest, No DOX to DOX, was performed. ***, *P* < 0.001; ns, not significant. All data are representative of two or more independent experiments.

To study the impacts of MARCH1 and -8 on IAV infection, 293T cell lines with stable DOX-inducible overexpression of MARCH1 and -8 proteins (with an N-terminal FLAG tag) or an irrelevant protein as a negative control (CTRL) were generated following lentiviral transduction. Flow cytometry 24 h after DOX induction, in the presence or absence of the proteasomal inhibitor MG132, confirmed intracellular expression of FLAG-tagged MARCH1 and -8 proteins (Fig. 1A), while immunoprecipitation and Western blotting confirmed the expression of proteins corresponding to the predicted molecular weights of MARCH1 (∼32 kDa) and MARCH8 (∼34 kDa) (Fig. 1B). Importantly, DOX-inducible expression of both MARCH1 and -8 resulted in potent downregulation of cell surface CD86 (Fig. 1C), confirming their functionality against a known transmembrane target (1).

### Overexpression of MARCH8 inhibits a late stage in the replication cycle of influenza A virus but does not inhibit human metapneumovirus.

**FIG 2:**
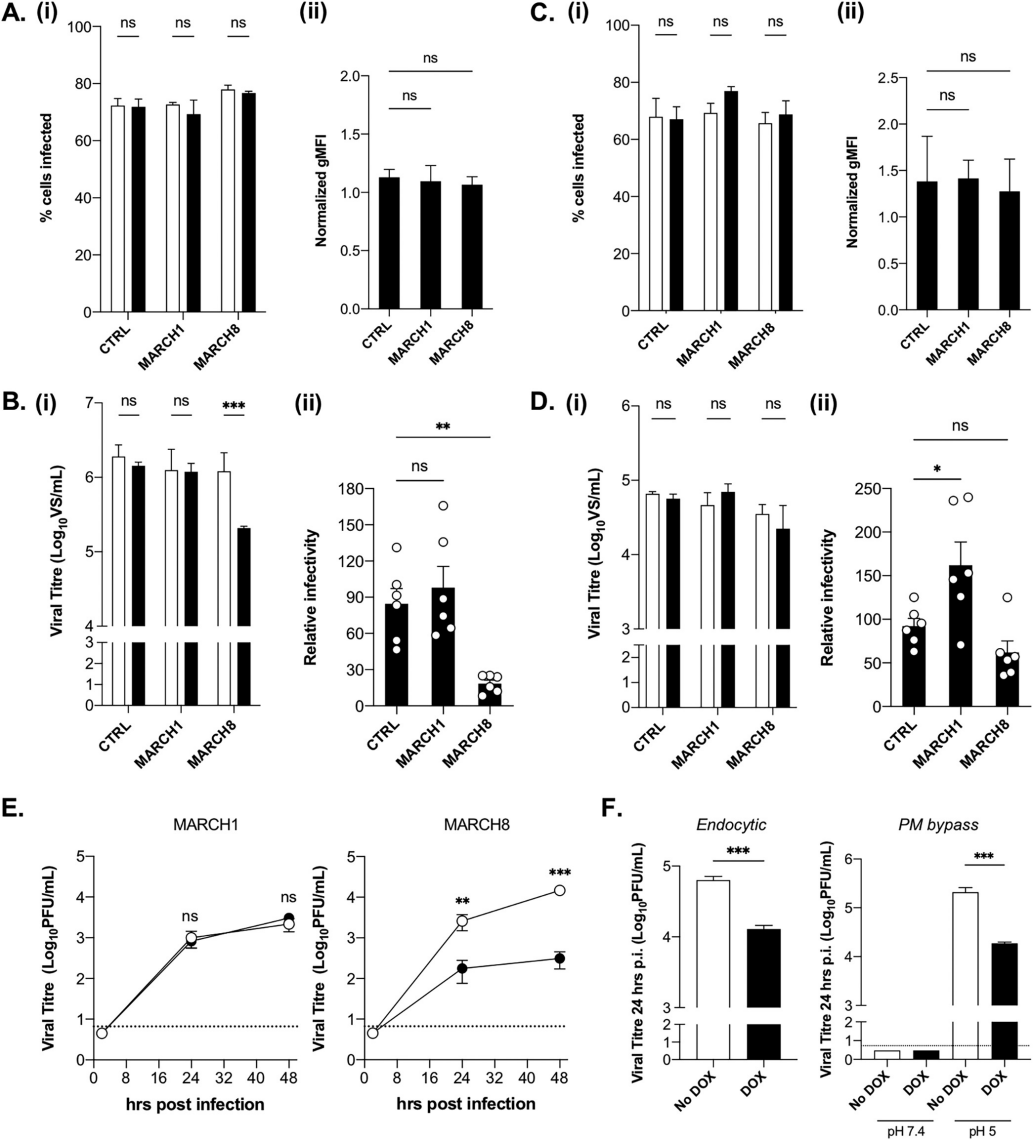
Overexpression of MARCH8, but not of MARCH1, mediates antiviral activity against a late stage of influenza A virus replication. Cells cultured in media (No DOX, white bars) or DOX induced for 24 h (DOX, black bars) were washed and infected with IAV strain Beij/89 (A and B) or hMPV strain CAN-97-83 (C and D). At 8 hpi (IAV, MOI = 2.5) (A) or 18 hpi (hMPV, MOI = 2) (C), the cells were washed, fixed, permeabilized, and stained with FITC-labeled MAbs to viral NP and N, respectively. (i) The percentages of virus-infected cells (left panel) were determined by flow cytometry. Two-way ANOVA using Bonferroni’s posttest, No DOX to DOX, was performed. (ii) gMFI values were normalized (DOX/No DOX) and plotted as means ± the standard deviations (SD). One-way ANOVA, using Dunnett’s posttest to CTRL, was performed. At 24 hpi (IAV, MOI = 5) (B) or 48 hpi (hMPV, MOI = 2) (D), the supernatants were harvested and clarified, and titers of infectious virus were determined by a ViroSpot assay using MDCK or Hep2 cells, respectively. (i) Mean virus titers from triplicate samples (± the SD) are shown. Two-way ANOVA, using Bonferroni’s posttest, No DOX to DOX, was performed. (ii) To determine the relative infectivity, titers from each cell line under the No DOX condition were set to 100%. One-way ANOVA, using Dunnett’s posttest to CTRL, was performed. (E) Cells cultured in media (No DOX, white circles) or DOX induced for 24 h (DOX, black circles) were infected with Beij/89 (MOI = 0.005) and cultured in the presence of exogenous trypsin (0.5 μg/ml) to enable multicycle replication. Virus titers in clarified supernatants from triplicate samples harvested at 2, 24, and 48 hpi were determined by a plaque assay. A two-tailed unpaired Student *t* test for No DOX versus DOX was performed at each time point. (F) Cells were infected with Beij/89 via conventional methods (endocytic route, MOI = 5) or via acid bypass assay (plasma membrane [PM] bypass, MOI = 25), and virus supernatants were collected at 24 hpi. Virus titers (mean ± SD) from triplicate samples are shown. A two-tailed unpaired Student *t* test for No DOX versus DOX was performed. *, *P* < 0.05; **, *P* < 0.01; ***, *P* < 0.001; ns, not significant. Dashed lines indicate the limit of detection. All data are representative of two or more independent experiments.

To assess the impact of inducible overexpression of MARCH1 or -8 on IAV infection, we first measured the expression of newly synthesized viral nucleoprotein (NP) since this would reveal any major impact of MARCH expression on preceding steps in the virus replication cycle (i.e., attachment, entry, fusion, transcription, and translation of viral and mRNA). Cells cultured in the presence (DOX) or absence (No DOX) of DOX were infected with IAV, and the intracellular NP expression was determined by flow cytometry at 8 h postinfection (hpi). No significant differences were observed in the percentages of NP^+^ cells (Fig. 2Ai) or in the normalized geometric mean fluorescence intensity (gMFI) of NP expression (Fig. 2Aii) after culture and infection in the presence or absence of DOX.

Next, to assess the impact of MARCH1 or -8 overexpression on late stages of infection (i.e., virus assembly, budding, and/or exit), we determined the titers of infectious particles released from virus-infected cells. For each 293T cell line, virus titers increased significantly between 2 hpi [all titers <4.8 log_10_(VS/ml)] and 24 hpi, which was consistent with productive virus replication (Fig. 2Bi). Although DOX-inducible expression of CTRL or MARCH1 proteins did not alter virus titers, MARCH8 expression reduced virus titers by ∼90% (Fig. 2Bii). Similar experiments using human metapneumovirus (hMPV) showed no significant reductions in the percentages of infected cells or in the normalized gMFIs of hMPV N protein expression at 18 hpi (Fig. 2C) or in virus titers at 48 hpi (Fig. 2Di) when comparing each cell line in No DOX versus DOX conditions in two independent experiments, although cells expressing MARCH1 did show a higher relative infectivity when these data were pooled (Fig. 2Dii). Thus, MARCH8 displays antiviral activity against IAV, but not hMPV, whereas MARCH1 did not display antiviral activity against either virus.

Given that DOX-inducible MARCH1 and -8 overexpression both downregulated CD86 (Fig. 1C), we next examined multicycle viral replication kinetics to determine whether subtle differences in IAV replication efficiency could be detected in the presence of MARCH1 or -8 overexpression. These experiments confirmed that only MARCH8 mediated potent inhibition of multicycle IAV growth (Fig. 2E). IAV infects cells via receptor-mediated endocytosis; however, at low pH, HA-mediated fusion can also be mediated at the plasma membrane. To confirm that MARCH8 restricts a late stage in IAV infection, we examined virus infection (early stages, 8 hpi) and growth (late stages, 24 hpi) after inducing IAV HA-fusion at the cell surface (“acid bypass” assay) (13). When IAV was delivered via conventional infection (endocytic entry) or by acid bypass assay (plasma membrane fusion), the virions released at 24 hpi from DOX-induced cells showed reduced virus titers compared to those released from cells cultured in No DOX conditions (Fig. 2F). Despite the reduced virus titers released at 24 hpi, inducible MARCH8 was associated with a higher percentage of total NP^+^ cells at 8 hpi (No DOX = 55.5% ± 3.12%, DOX = 76.6% ± 0.82% total NP^+^ cells at 8 hpi). Thus, MARCH8 restricts IAV at a late stage in the virus replication cycle irrespective of the route of infectious virus entry.

### MARCH8 expression is associated with reduced virus titers and less hemagglutination and NA enzymatic activity against a range of human influenza viruses.

**FIG 3:**
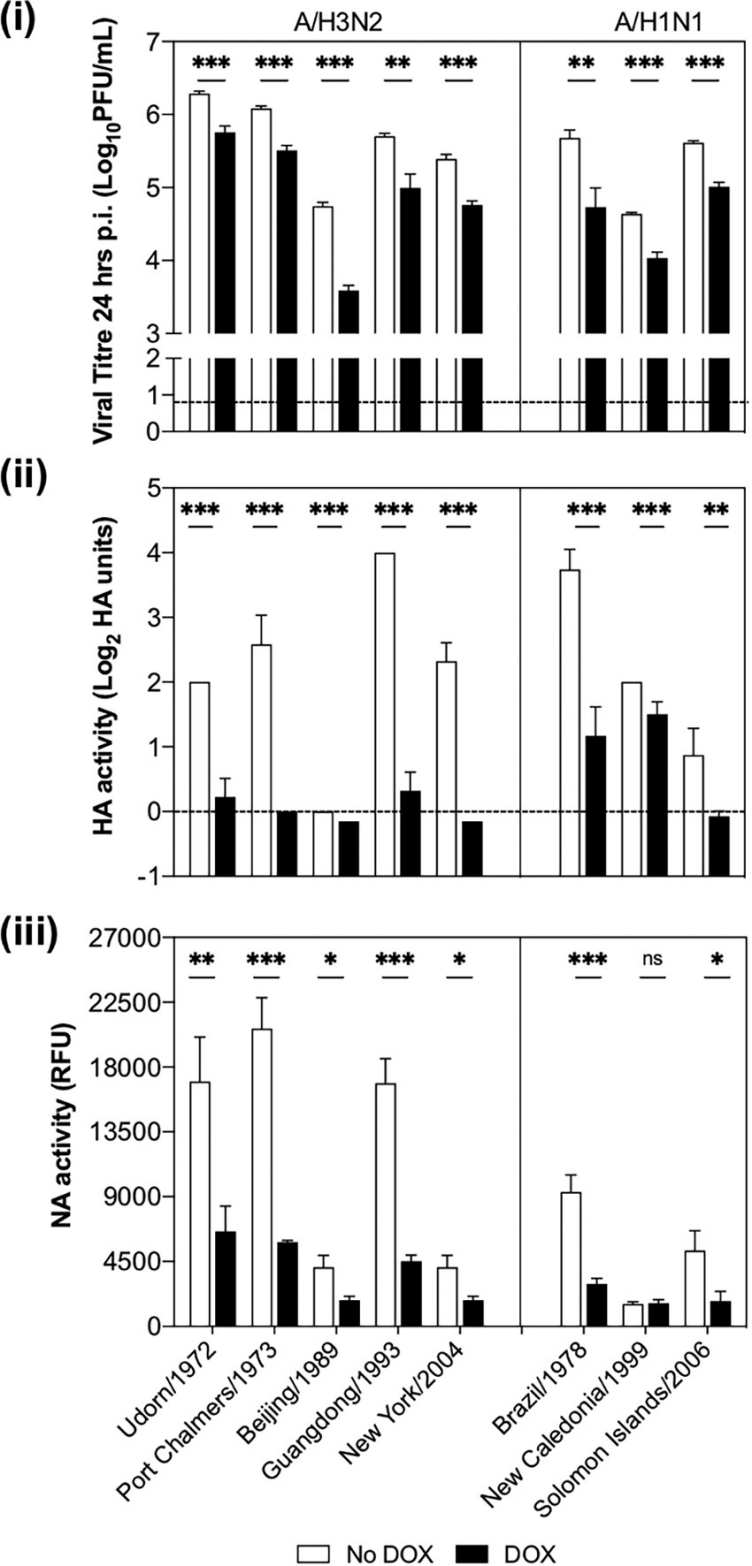
MARCH8 overexpression is associated with antiviral activity against a range of human influenza viruses. Cells cultured for 24 h (No DOX, white bars) or DOX induced (DOX, black bars) were infected with different IAV strains (MOI = 5 PFU/cell) and washed, and virus titers in clarified supernatants were determined at 24 hpi (i). Mean virus titers ± the SD from triplicate samples are shown. The dashed line indicates the limit of detection. (ii) Hemagglutination assay. Mean HA units ± the SD from triplicate samples are shown. The dashed line indicates the limit of detection. (iii) NA activity. A two-tailed unpaired Student *t* test for No DOX versus DOX was performed. *, *P* < 0.05; **, *P* < 0.01; ***, *P* < 0.001; ns, not significant. All data representative of two or more independent experiments.

Next, we assessed the ability of MARCH8 to mediate antiviral activity against a range of seasonal A/H3N2 and A/H1N1 viruses. As seen in Fig. 3i, DOX-inducible MARCH8 expression resulted in a significant reduction in titers of virus released from cells infected with all IAV strains tested. Since the viral HA and NA proteins modulate IAV entry and release, we also assessed the ability of virions released from 293T cells in the presence or absence of MARCH8 to agglutinate erythrocytes (a function of the viral HA) or cleave MUNANA substrate (a function of the viral NA). Virions released in the presence of MARCH8 showed reduced hemagglutination (Fig. 3ii) and NA enzymatic activity (Fig. 3iii) for all of the virus strains tested, with the exception of A/New Caledonia/1999, which showed very low NA activity in both the presence and the absence of DOX. Thus, MARCH8 expression was associated with reduced titers of infectious virus, as well as less HA and NA activity, across a range of IAV strains and subtypes, though it should be noted that the potency of MARCH8 antiviral activity did vary between virus strains.

### Induction and knockdown of endogenous MARCH8 expression in the context of IAV infection.

**FIG 4:**
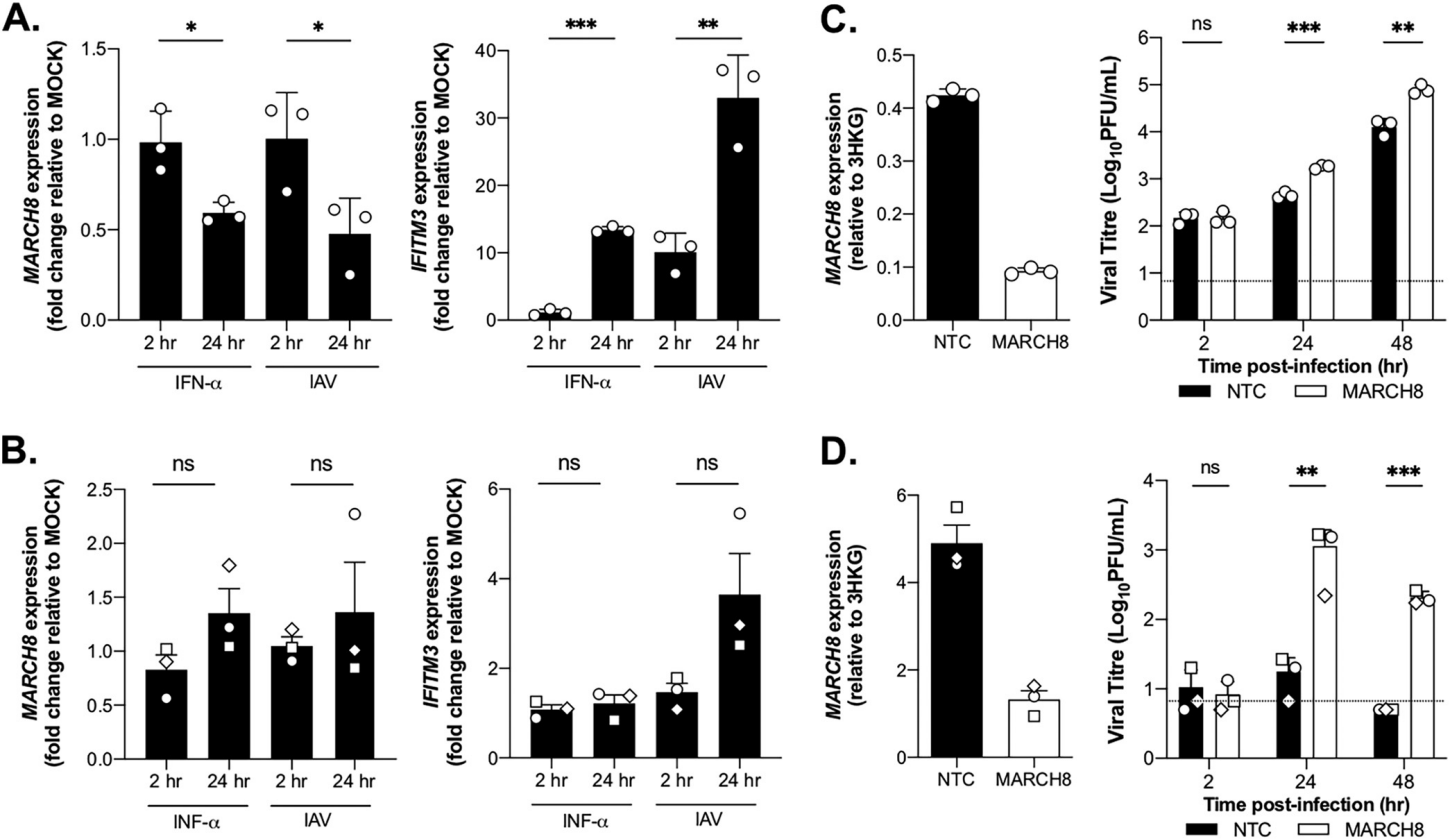
Endogenous MARCH8 expression is not induced in 293T cells or hMDMs by IFN or IAV, but knockdown does result in increased IAV replication. 293T cells (A) or hMDMs (B) were treated with 500 U/ml IFN-α, infected with IAV (Beij/89, MOI 10), or left untreated (mock). At 2 and 24 h, the total RNA was extracted, and RT-qPCR was performed to determine the expression of mRNA for MARCH8 and IFITM3, relative to three housekeeping genes. The data are expressed as fold changes in mRNA levels relative to mock-treated samples. A two-tailed unpaired Student *t* test for 2 h versus 24 h was performed. 293T cells (C) or hMDMs (D) were transfected with siRNAs specific for MARCH8 or nontargeting control (NTC) and 72 h later infected with Beij/89 (293T cells [MOI = 0.01] and hMDMs [MOI = 2.5]) in the presence of exogenous trypsin. Quantitation of MARCH8 mRNA in infected cell lysates was performed by RT-qPCR at 2 hpi (left panel). At 2, 24, and 48 hpi, the titers of infectious virus were determined by a plaque assay (right panel). A two-tailed unpaired Student *t* test for NTC versus MARCH8 at each time point was performed. *, *P* < 0.05; **, *P* < 0.01; ***, *P* < 0.001; ns, not significant. The data are representative of two independent experiments.

Given that MARCH8 overexpression was associated with potent anti-IAV activity, we next investigated whether endogenous MARCH8 could also inhibit IAV. Since the levels of endogenous MARCH8 were too low for robust quantification by Western blotting, RT-qPCR was used to detect the presence of MARCH8 mRNA. First, we determined endogenous versus inducible MARCH8 in 293T cells (endogenous = 0.383 ± 0.025 and 0.410 ± 0.113 versus inducible = 0.15 ± 0.017 and 25.920 ± 1.170 in No DOX and DOX samples, respectively). Next, we showed that MARCH8 mRNA expression in 293T cells (Fig. 4A) was reduced between 2 and 24 h after (i) exposure to type I interferon (IFN) or (ii) infection with IAV, whereas IFN-regulated gene IFITM3 was strongly upregulated by both type I IFN and by IAV infection; it should be noted that previous studies reported rapid (0.5 h) induction of MARCH8 by type I IFNs in 293T cells, which returned to baseline levels within 2 to 4 h (8). Similar results were seen using human monocyte-derived macrophages (hMDMs) (Fig. 4B), which expressed notably higher levels of MARCH8, consistent with previous studies (11, 14). Since hMDMs were prepared by culture in the presence of IFN-γ, it was not surprising to see no further induction after type I IFN treatment.

Next, 293T cells or hMDMs (three independent donors) pretreated with siRNA for 72 h were infected with IAV, and clarified cell supernatants (for plaque assay) and cell lysates (for MARCH8 RT-qPCR) collected at 2, 24, and 48 hpi. RT-qPCR confirmed efficient knockdown of MARCH8 mRNA expression in both cell types (Fig. 4C and D, left panels). Compared to nontargeting control (NTC)-treated cells, MARCH8-specific small interfering RNA (siRNA) resulted in a modest but significant increase in virus titers released from 293T cells at 24 and 48 hpi (Fig. 4C, right panel). Moreover, virus titers released from hMDMs increased 10- to 100-fold at 24 and 48 hpi after MARCH8 knockdown (Fig. 4D, right). Together, these data confirm that endogenous MARCH8 protein also displayed antiviral activity toward IAV.

### MARCH8 restriction of IAV is associated with a reduced infectivity per particle and decreased incorporation of viral glycoproteins into virions.

**FIG 5:**
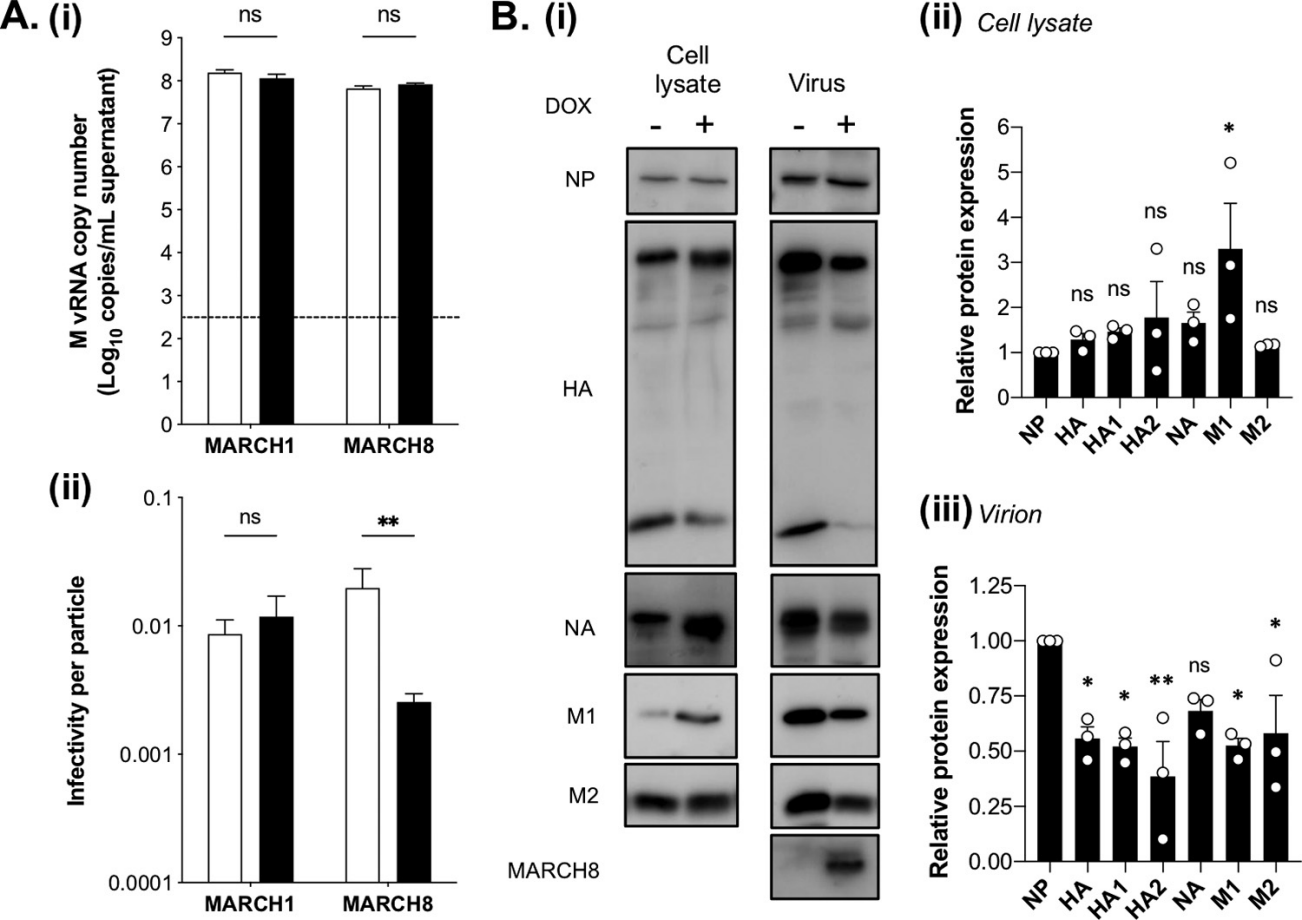
MARCH8 restriction of IAV is associated with a reduced infectivity per particle and decreased incorporation of viral glycoproteins into virions. Cells cultured in media (No DOX, white bars) or DOX induced for 24 h (DOX, black bars) were infected with Beij/89 (MOI = 5). (A) At 24 hpi, vRNA was extracted from clarified supernatants, and the M gene copy number was determined by RT-qPCR. (i) Mean (± SD) copy numbers (triplicate samples, *n* = 2 independent experiments). The dashed line indicates the limit of detection. (ii) The infectivity per particle is the ratio between the viral titer and the M vRNA copy number. For panels i and ii, the mean (± the SD) values from triplicate samples are shown. A two-way ANOVA, using the Bonferroni’s posttest, was performed. (B) At 24 hpi, the cells were lysed, whereas the supernatants were harvested and concentrated by ultracentrifugation, before both were subjected to SDS-PAGE under reducing conditions, followed by Western blotting to detect viral proteins (NP, HA, NA, M1, and M2) and MARCH8 expression with anti-FLAG MAb. (i) Representative data from one of three experiments are shown. (ii) After densitometry, viral protein expression was normalized to the viral NP. The data show the means (± the SD) from three experiments. One-way ANOVA, using Dunnett’s posttest to NP, was performed.

To determine whether the antiviral activity of overexpressed MARCH8 toward IAV corresponded to a decrease in the overall number of viral particles released or to a reduced infectivity per particle, we used qPCR to quantify the amount of viral RNA (vRNA) in cell-free supernatants from infected cells. At 24 hpi, M gene vRNA was detected at a similar copy number in the presence or absence of MARCH1 or -8 overexpression (Fig. 5Ai). Similar results were obtained when we quantified the HA gene vRNA produced from the same cells (data not shown). The ratio between the viral titer (plaque assay) and the total number of particles released (qPCR) (15) confirmed that induction of MARCH8, but not MARCH1, led to a reduction in the infectivity per particle of virions released from IAV-infected cells (Fig. 5Aii).

Differences in the relative proportion or the absolute amount of specific viral proteins in a packaged virion could contribute to overall reduction in IAV infectivity. Reduced incorporation of Env into HIV-1 (14) or VSV-G, EboV-GP, and SARS-CoV-2 S glycoproteins into pseudotyped particles (8) correlated with reduced infectivity of virions released from MARCH8-expressing cells. As seen in Fig. 5Bi, IAV proteins (NP, HA, NA, M1, and M2) were detected in cell lysates from No DOX- or DOX-treated cells expressing inducible MARCH8. To facilitate comparison between virions released from cells that do or do not overexpress MARCH8, we normalized the data to an internal RNA-binding protein of the virion (NP), given that similar levels of viral genome were detected in the supernatants from infected cells in the presence or absence of MARCH8 (Fig. 5A). After normalization, densitometry indicated significantly higher levels of M1 protein in cells overexpressing MARCH8 (Fig. 5Bii) and a trend toward increased levels of HA, NA, and M2 viral proteins, although these findings were not significant. Virions released from cells overexpressing MARCH8 expressed reduced amounts of HA, M1, and M2 proteins relative to NP, with a trend toward reduced NA levels (Fig. 5Biii). No accumulation of HA_0_ was noted in lysates, consistent with no major differences in intracellular HA cleavage. Moreover, confocal microscopy confirmed no major differences in HA expression patterns in cells infected with A/Beijing/353/89 (Beij/89) at 8 hpi in the presence or absence of MARCH8 (data not shown). Western blot analysis indicated that MARCH8 was also incorporated into virions released from cells overexpressing MARCH8 after IAV infection, although it should be noted that this could represent MARCH8 in microvesicles which had been concentrated along with the virions.

### IAV glycoprotein expression on the surfaces of infected cells is not decreased by MARCH8 expression, and the HA, NA, and M proteins are not essential targets for MARCH8-mediated ubiquitination.

**FIG 6:**
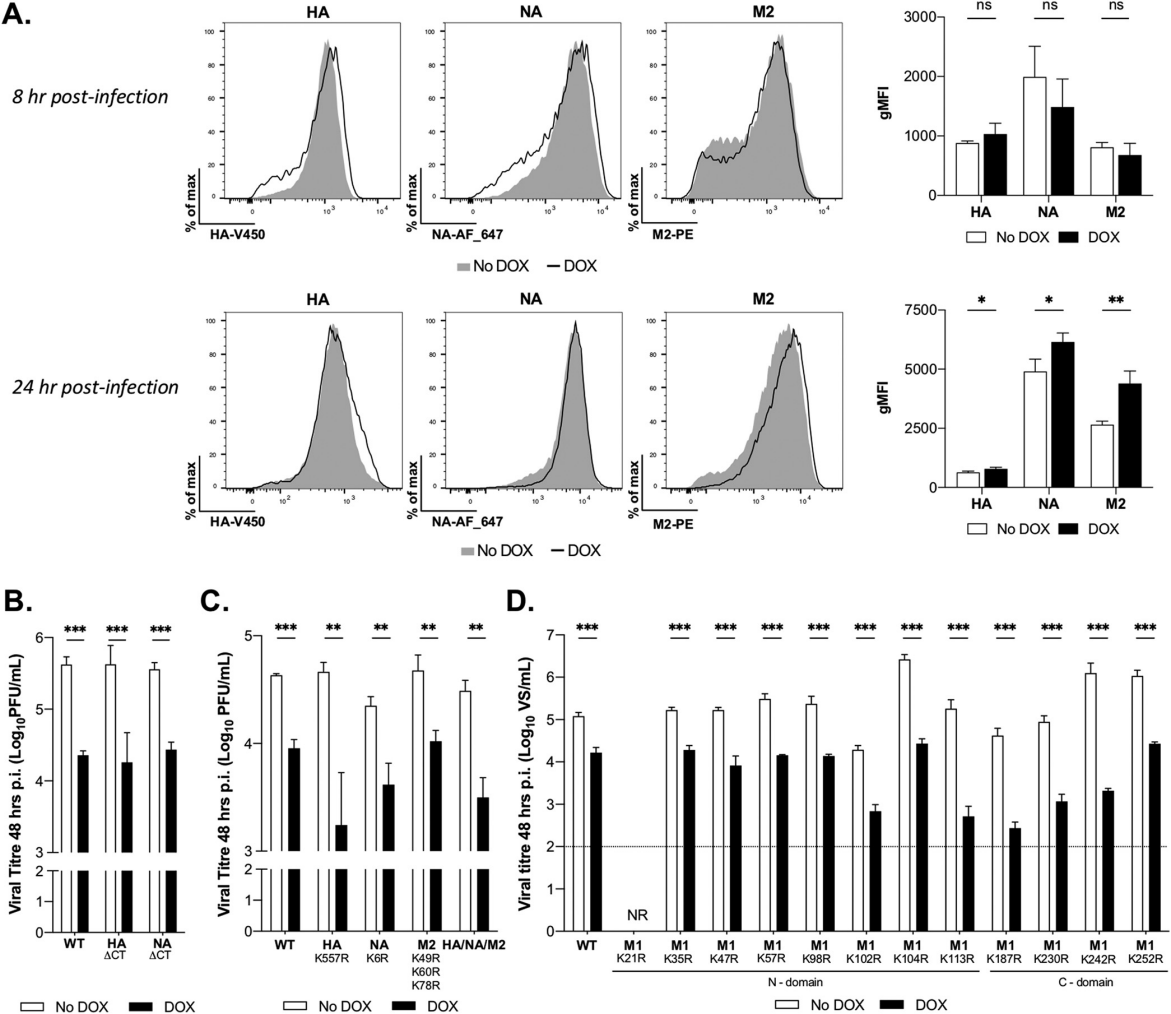
Inhibition of IAV infectivity by MARCH8 is not associated with reduced viral protein expression from the surfaces of infected cells and does not involve the recognition of lysine residues in the CTs of viral HA, NA, or M proteins. (A) Cells cultured in media (No DOX, white bars) or DOX induced for 24 h (DOX, black bars) were infected Beij/89 (MOI = 1 PFU/cell). At 8 or 24 hpi, the cells were stained with specific antibodies to determine the cell surface expression of HA, NA, or M2 and then fixed, permeabilized, and stained for intracellular NP. The surface expression of HA, NA, or M2 on NP^+^ cells was determined by flow cytometry. Representative histograms (left panels) and mean (± the SD) gMFI values (right panel) from triplicate samples are shown. (B to D) Cells cultured in media (No DOX, white bars) or DOX induced for 24 h (DOX, black bars) were infected with WT or mutant viruses (MOI = 0.01 PFU/cell) in the presence of exogenous trypsin, and virus titers in clarified supernatants were determined at 48 hpi. Mean virus titers from triplicate samples (± the SD) are shown. Site-directed mutagenesis was used to generate viruses lacking the CT of HA (HA ΔCT) or NA (NA ΔCT) (B) or to introduce lysine-to-arginine (K-to-R) mutations in the CTs of HA, NA, and M2 (C). Recombinant viruses carrying wild-type (WT) or mutant versions of individuals (HA K557R; NA K6R), or all K residues in the CT of one protein (M2 K49R, K60R, and K78R), or all K residues in the CT of three viral proteins (HA/NA/M2) were generated by reverse genetics. Virus titers were determined by plaque assay. (C) Site-directed mutagenesis was used to introduce individual K-to-R mutations into the M1. Recombinant viruses carrying WT or mutant versions of M1 were generated by using reverse genetics. NR, virus not rescued. Virus titers were determined by a ViroSpot assay. The dashed line shows the limit of detection. All data are representative of two independent experiments (i) and a two-tailed unpaired Student *t* test for No DOX versus DOX (ii). *, *P* < 0.05; **, *P* < 0.01; ***, *P* < 0.001; ns, not significant.

Human (8, 14, 16) and mouse (9) MARCH8 reduce the expression of a range of viral glycoproteins from the surface of infected cells, thereby reducing their incorporation into virions. Surprisingly, we detected no significant differences in the cell surface expression of HA, NA, or M2 proteins in IAV-infected cells in presence or absence of inducible MARCH8 at 8 hpi, a relatively early time point in the IAV replication cycle (Fig. 6A). At 24 hpi, a time when virions are being released from IAV-infected cells, we actually detected a modest but significant increase in cell surface expression of HA, NA, and M2 in the presence of MARCH8. These results indicate that MARCH8 does not interfere with the synthesis and transport of viral HA, NA, or M2 to the surface of IAV-infected cells. Thus, the antiviral activity of MARCH8 against IAV at a late stage of the replication cycle cannot be attributed to a reduction in the expression of viral glycoproteins on the surfaces of infected cells.

For VSV-G, lysine (K) residues in its cytoplasmic tail (CT) are targeted by the RING-CH domain of MARCH8, resulting in ubiquitin-dependent downregulation of VSV-G expression and reduced incorporation into pseudotyped particles (8, 10). The CTs of IAV HA/NA proteins each contain a single K residue, while the CT of the IAV M2 protein contains three. Reverse genetics was used to generate recombinant IAV with the entire CT of either HA or NA deleted, and both viruses remained sensitive to MARCH8-mediated restriction (Fig. 6B). Next, we generated viruses wherein K residues were exchanged to arginine (R) residues in the CTs of the HA (K557R), NA (K6R), and M2 (K49R, K60R, K78R) proteins, separately or in combination (HA/NA/M2). All recombinant IAVs were viable, and viruses carrying mutations in the CTs of HA (K557R), NA (K6R), and M2 (K49R, K60R, and K78R) or simultaneously in all three viral proteins (HA/NA/M2) were all restricted by MARCH8, although the potency of inhibition did vary between viruses (Fig. 6C). In addition to HA, NA, and M2 expression at the plasma membrane, M1 is recruited to sites of IAV assembly and budding, where it interacts with the inner leaflet of the plasma membrane and the CTs of HA and NA (17–19). There are eight K residues in the N-terminal domain of M1 thought to interact with the plasma membrane and four residues in the C-terminal domain. We generated single K-to-R mutants for 11 M1 residues; however, mutant viruses expressing K21R could not be generated. Despite some variation in virus titers released from uninduced 293T cells after infection with different IAV M1 mutants, all remained sensitive to restriction by MARCH8 (Fig. 6D). Interestingly, some of the mutations in M1 actually appeared to result in viruses with an increased susceptibility to MARCH8 restriction (Fig. 6C).

### A functional RING-CH domain and a tyrosine-based motif in the C-terminal cytoplasmic tail of MARCH8 are essential for antiviral activity against IAV.

**FIG 7:**
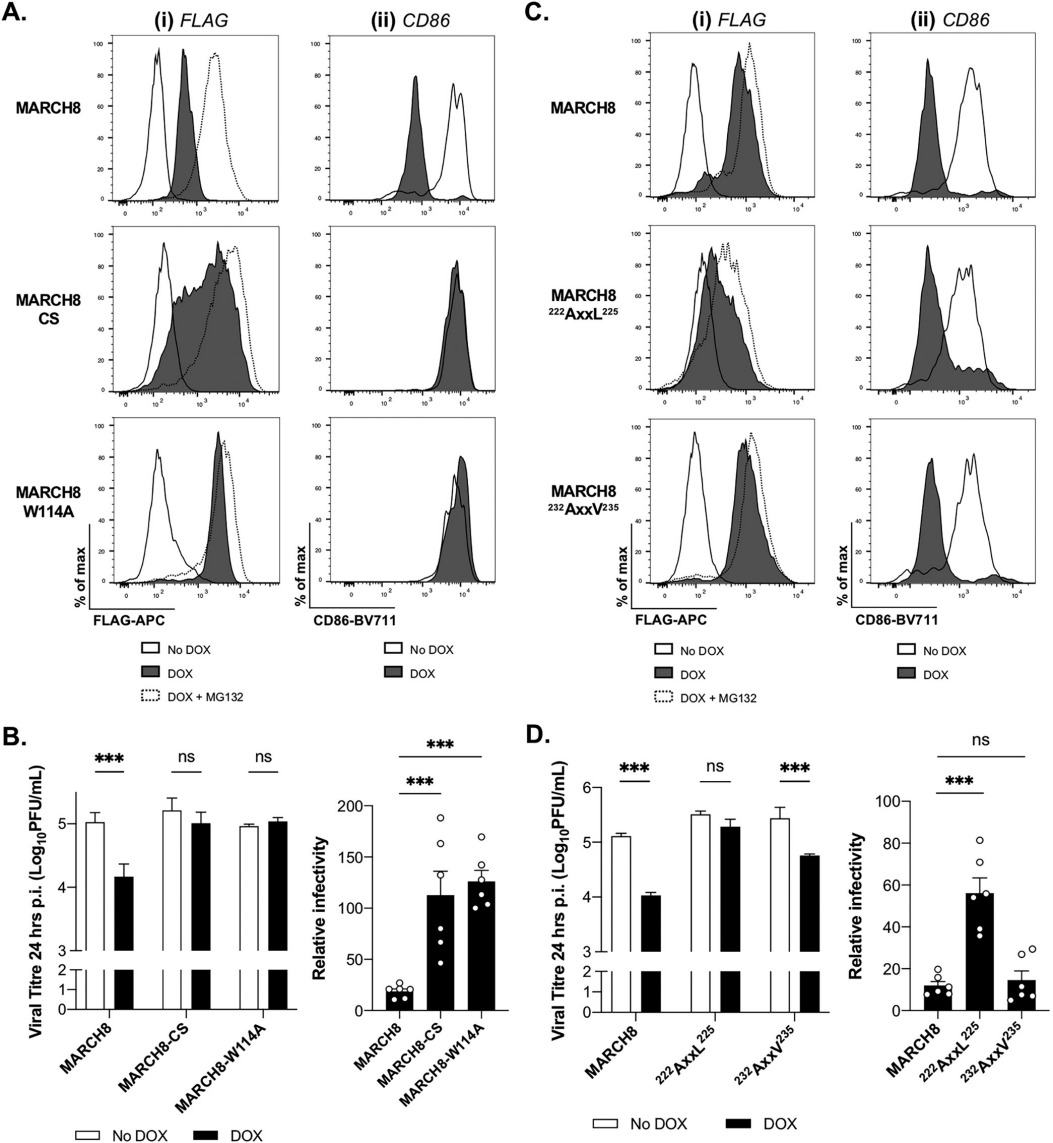
MARCH8-mediated inhibition of IAV requires E3 ligase activity and a tyrosine-based motif in the C-terminal cytoplasmic tail of MARCH8. Stable cell lines with DOX-inducible expression of WT MARCH8 or MARCH8 RING-CH mutants MARCH8-CS or MARCH8-W114A (A and B) or MARCH8 tyrosine motif mutants MARCH8-^222^AxxL^225^ or MARCH8-^232^AxxV^235^ (C and D) were generated. (A and C, subpanels i) Cells cultured for 24 h in media (No DOX), DOX induced (DOX), or DOX induced in the presence of MG132 (DOX+MG132) were fixed, permeabilized, and stained with anti-FLAG-APC MAb. Representative histograms are shown. (A and C, subpanels ii) Cells were transfected with a plasmid encoding CD86 and ZsGreen fluorescent protein. At 12 h posttransfection, MARCH1 and -8 expression was induced with DOX and, 24 h later, the cells were stained for cell surface CD86. Representative histograms are shown. (B and D) Cells cultured for 24 h in media (No DOX, white bars) or DOX induced for 24 h (DOX, black bars) were infected with Beij/89 (MOI of 5 PFU/cell) and washed, and virus titers in clarified supernatants were determined at 24 hpi. Mean virus titers from triplicate samples (± the SD) are shown. Two-way ANOVA, using Bonferroni’s posttest for No DOX versus DOX, was performed. For the relative infectivity, titers from each cell line in the No DOX condition were set to 100%. One-way ANOVA, using Dunnett’s posttest to WT MARCH8, was performed. All data are representative of two independent experiments (i), and the significance is indicated (*, *P* < 0.05; **, *P* < 0.01; ***, *P* < 0.001; ns, not significant) (ii).

Mutations in the RING domain known to disrupt E3 ligase activity have been shown to abrogate the ability of MARCH8 to decrease viral glycoprotein expression and/or infectivity of other viruses (8, 14, 16). Therefore, 293T cell lines with DOX-inducible expression of the E3 ligase mutants MARCH8-CS (20) or MARCH8-W114A (21) were generated. First, we confirmed expression levels of each RING-CH domain mutant relative to parental MARCH8 after DOX induction (Fig. 7Ai). To validate each mutation, we confirmed that expression of MARCH8, but not MARCH8-CS or MARCH8-W114A, resulted in the downregulation of cell surface CD86 (Fig. 7Aii). As expected, the induced expression of either parental or E3 ligase mutants of MARCH8 did not affect early events in the virus replication cycle, as determined by assessing NP^+^ cells at 8 hpi under No DOX versus DOX conditions (data not shown). However, in contrast to MARCH8, overexpression of either MARCH8-CS or MARCH8-W114A was not associated with reduced virus titers at 24 h p.i. (Fig. 7B). These data confirm that a functional RING-CH domain is essential for the anti-IAV activity of MARCH8.

The cytoplasmic ^222^YxxL^225^, but not the ^232^YxxV^235^ motif of MARCH8, is critical for the inhibition of HIV-1, likely via interactions between the tyrosine residue in the C-terminal CT of MARCH8 with adaptor proteins resulting in intracellular retention of HIV-1 Env in the *trans*-Golgi network (TGN) (10). Therefore, we generated 293T cell lines with DOX-inducible expression of tyrosine motif mutants of MARCH8 (designated ^222^AxxL^225^ or ^232^AxxV^235^). After DOX induction, intracellular staining indicated that MARCH8-^222^AxxL^225^, but not MARCH8-^232^AxxV^235^, was expressed at reduced levels compared to parental MARCH8 (Fig. 7Ci), although both mutants still downregulated CD86 (Fig. 7Cii). The cell lines with inducible expression of parental, ^222^AxxL^225^, or ^232^AxxV^235^ MARCH8 were all infected to similar levels by IAV, as detected by viral NP expression at 8 hpi (data not shown). However, only cells expressing parental or ^232^AxxV^235^ MARCH8 displayed antiviral activity toward IAV (Fig. 7D). Thus, mutation of the cytoplasmic ^222^YxxL^225^ motif of MARCH8 resulted in reduced intracellular levels of MARCH protein and also abrogated anti-IAV activity.

### The N-terminal cytoplasmic domain of MARCH8 is essential for IAV restriction.

Of the MARCH proteins, MARCH1 and -8 show the highest sequence conservation across the entire protein (65%) with up to 89% in the RING-TM1/2 domains (reviewed in reference 6). The N-terminal cytoplasmic domain (N-CT) is the most variable region between MARCH1 and -8, and the stability and turnover of mouse MARCH1 is controlled by sequence elements in its N-CT (22). To investigate the role of the N-CT in differential restriction of IAV we generated 293T cells with inducible expression of MARCH1 or -8 with their N-CT domain (i) deleted (MARCH1_ΔN-CT and MARCH8_ΔΝ-CT, respectively) or (ii) swapped (i.e., MARCH8 containing the N-CT of MARCH1 [MARCH8_M1 N-CT] and MARCH1 containing the N-CT of MARCH8 [MARCH1_M8 N-CT]).

**FIG 8:**
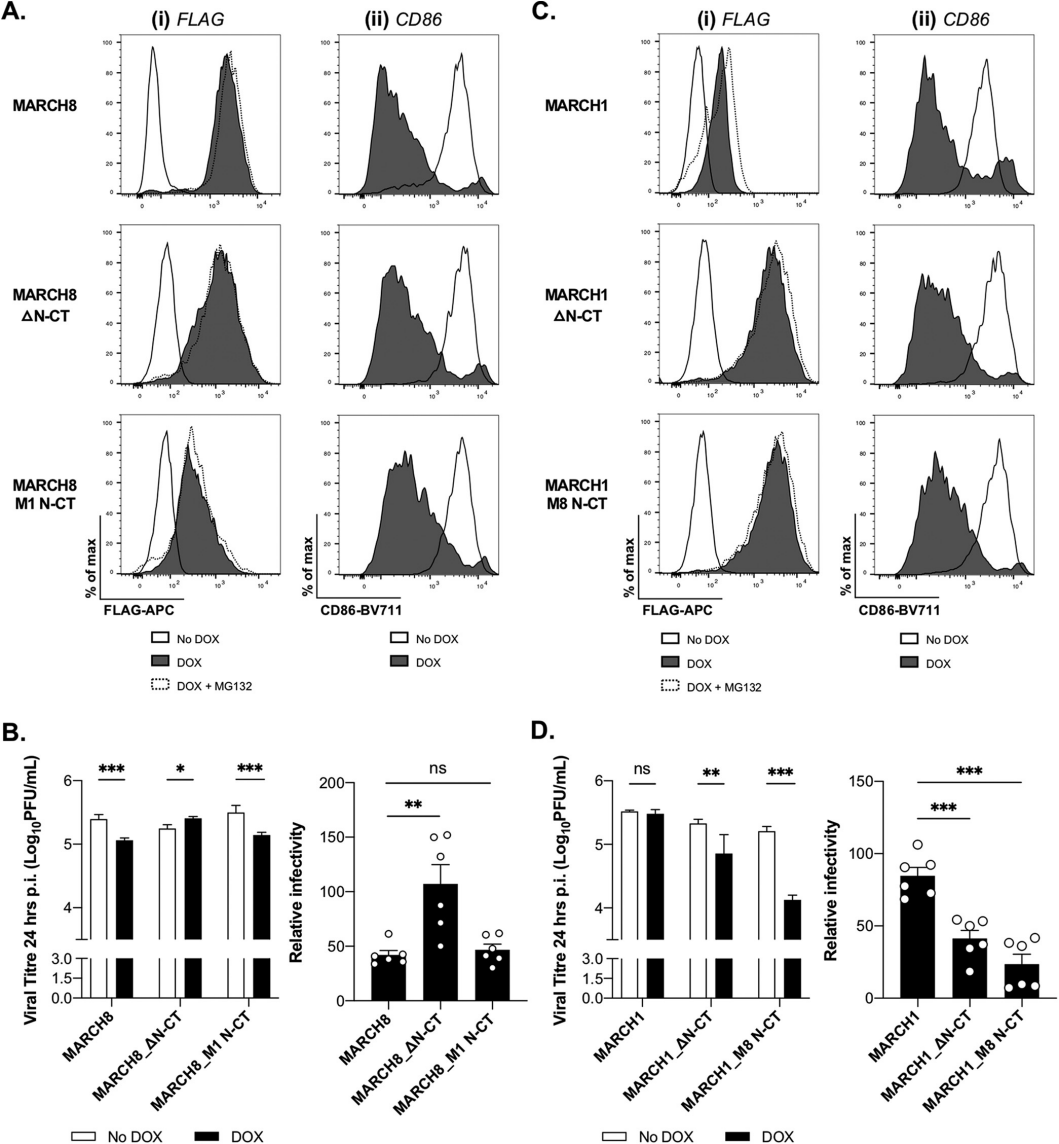
The N-terminal cytoplasmic tail (N-CT) of MARCH8 is critical for anti-IAV activity, whereas removal or replacement of the MARCH1 N-CT resulted in the acquisition of IAV restriction. Cell lines with DOX-inducible expression of parental MARCH8 or MARCH8 with N-CT deletion (MARCH8_ΔN-CT) or substitution for the N-CT of MARCH1 (MARCH8_M1 N-CT) (A and B) or parental MARCH1 or MARCH1 with N-CT deletion (MARCH1_ΔN-CT) or substitution for the N-CT of MARCH8 (MARCH1_M8 N-CT) (C and D) were generated. (A and C, subpanels i) Cells cultured for 24 h in media (No DOX), DOX induced (DOX), or DOX induced with MG132 added for the last 4 h (DOX+MG132) were fixed, permeabilized, and stained with anti-FLAG-APC MAb. Representative histograms are shown. (A and C, subpanels ii) Cells were transfected with a plasmid encoding CD86 and ZsGreen fluorescent protein. At 12 h posttransfection, MARCH1 and -8 expression was induced with DOX and, 24 h later, the cells were stained for cell surface CD86. Representative histograms are shown. (B and D) Cells cultured in media (No DOX, white bars) or DOX induced for 24 h (DOX, black bars) were infected with Beij/89 (MOI = 5 PFU/cell) and washed, and virus titers in clarified supernatants were determined at 24 hpi. Two-way ANOVA, using Bonferroni’s posttest for No DOX versus DOX, was performed. For the relative infectivity, titers from each cell line in No DOX condition were set to 100%. One-way ANOVA, using Dunnett’s posttest to WT MARCH8 (B) or WT MARCH1 (C), was performed. All data are representative of two independent experiments (i), and the significance is indicated (*, *P* < 0.05; **, *P* < 0.01; ***, *P* < 0.001; ns, not significant) (ii).

Deletion of MARCH8 N-CT did not have a major impact on MARCH8 protein expression levels (Fig. 8Ai) or its ability to downregulate CD86 (Fig. 8Aii); however, this mutant did not display antiviral activity toward IAV (Fig. 8B). Substituting the MARCH1 N-CT into MARCH8 (MARCH8_M1 N-CT) reduced the levels of MARCH protein expression (Fig. 8Ai); however, this protein still downregulated CD86 (Fig. 8Aii) and retained anti-IAV activity (Fig. 8B). These findings indicate that MARCH8 N-CT is a critical determinant of anti-IAV activity. In contrast, deletion of the MARCH1 N-CT resulted in increased MARCH protein expression (Fig. 8Ci), correlating with acquisition of anti-IAV activity (Fig. 8D) and swapping the MARCH8 N-CT into MARCH1 further enhanced the potency of anti-IAV activity (Fig. 8D) without impacting levels of MARCH protein expression (Fig. 8Ci). Despite these differences, the parental MARCH1, ΔN-CT, and MARCH1_M8 N-CT proteins all downregulated CD86 efficiently (Fig. 8Cii). Although deletion or substitution of the MARCH1 N-CT resulted in increased levels of MARCH protein expression, this does not appear to be the sole determinant of anti-IAV activity given (i) MARCH8 and MARCH8−ΔN-CT are expressed at similar levels, but only MARCH8 restricts IAV, and (ii) the increased potency of IAV restriction by MARCH1_M8 N-CT compared to MARCH1 N-CT deletion, despite similar expression levels.

## DISCUSSION

Virions produced in the presence of overexpressed MARCH8 were significantly less infectious than those from uninduced cells or from cells that overexpressed E3 ligase mutants of MARCH8. For HIV-1 and viruses pseudotyped with other viral glycoproteins, the reduced expression of viral glycoproteins at the cell surface is a key determinant of MARCH8-mediated antiviral activity (8, 9, 14, 16). However, after IAV infection, there was no MARCH8-dependent deficit in the cell surface expression of HA, NA, or M2 viral proteins. These findings suggest that MARCH8 might impact IAV assembly and/or budding rather than the transport and turnover of viral proteins at the plasma membrane. During virus assembly and budding, HA and NA accumulate in lipid rafts and M1 interacts with the vRNPs, with the CT tails of HA and NA, and with the inner leaflet of the plasma membrane (reviewed in reference 12). Although often associated with targeting proteins for internalization with or without degradation, ubiquitination by MARCH proteins can also provide platforms to promote intracellular protein-protein interactions. For example, MARCH8-driven polyubiquitination of the nonstructural protein 2 (NS2) of HCV was required for the recruitment of ESCRT machinery and subsequent envelopment of HCV (23). Given that IAV assembly and budding are highly coordinated processes involving multiple host and viral proteins, future studies will investigate whether MARCH8 impacts protein localization and/or interactions at sites of assembly and budding in IAV-infected cells. This could provide a plausible explanation for MARCH8-mediated anti-IAV activity.

When viral glycoproteins are targeted directly by MARCH8, antiviral activity is associated with recognition of K residues in the CT (e.g., VSV-G [8, 10] and MLV p15E [9]). However, MARCH8-mediated downregulation of HIV-1 (Env), EboV (GP), and SARS-CoV-2 (S) expression also occurred with deletion mutants lacking the CT domain of each glycoprotein (8, 10). Recombinant IAV generated by reverse genetics indicated that K residues in the CT of HA, NA, or M2 viral proteins were likely not to be the primary determinants of MARCH8 anti-IAV activity in our system. Furthermore, single K-to-R substitutions in 11 of the 12 K residues distributed throughout the viral M1 protein did not abrogate MARCH8 anti-IAV activity, noting that we could not generate a viable K21R mutant, nor did we generate mutant viruses with multiple K-to-R mutations in M1, as performed for HA, NA, and M2, and cannot exclude that multiple K residues in M1 might be targeted. Addition of ubiquitin to specific substrates generally requires K residues as acceptor sites for ubiquitination. Although cysteine (C), serine (S), and threonine (T) residues can be used by some viral E3 ligases, there is no evidence to date to support this for mammalian MARCH8 (reviewed in references 24, 25, and 26). The Beij/89 and A/Udorn/307/72 (Udorn/72) viruses used in our studies both express 2C in HA CT, no C/S/T residues in the NA CT and 1C/1T, and five or four S residues in the M2 CT, respectively. Aside from HA, NA, M1, and M2 proteins, it is also possible that MARCH8 targets other IAV proteins for ubiquitination, including possibly the PA, PB1, or PB2 proteins of the polymerase complex, noting that our Western blot did not detect a major deficit in viral NP levels in infected cells or released virions. Although other viral glycoproteins have been directly and indirectly targeted and downregulated by MARCH8, our studies suggest that this does not occur in IAV-infected cells. As reported for all other viruses to date, anti-IAV activity is dependent on the E3 ligase activity of MARCH8, although our studies indicate that K residues in the CT of HA, NA, or M2 or in the viral M1 protein do not appear to be a major determinant of MARCH8-mediated antiviral activity toward IAV.

Aside from VSV-G and MLV p15E, MARCH8 does not appear to directly target the envelope glycoproteins of other viruses to downregulate expression. This suggests that MARCH8 instead targets cellular proteins that control turnover of a broad range of viral glycoproteins. Indeed, the results presented here argue that MARCH8-mediated ubiquitination of a host protein is likely to be important for MARCH8-mediated restriction of IAV. Multiple host proteins regulate the late stages of IAV infection, including trafficking of vRNPs and viral glycoproteins to the plasma membrane, as well as virion assembly and budding (reviewed in reference 27). Host proteins such as RACK1, CD81, and F1Fo ATPase have been implicated in IAV assembly and budding, playing roles in the induction of membrane curvature and in “pinching-off” of budding particles (28–30). Of interest, CD81 is targeted by MARCH8 but not by MARCH 1 (7), and only MARCH8 potently restricted IAV infectivity in our studies. Given that host proteins are major components of the IAV virion (31), mass spectrometry of released virions and/or plasma membrane preparations from IAV-infected cells expressing MARCH8 versus MARCH1 (or MARCH8 versus E3 ligase mutants of MARCH8) represents a logical step toward identifying the cellular protein targets associated with the antiviral activity of MARCH8 toward IAV and possibly other viruses.

MARCH8-mediated downregulation of cell surface-expressed HIV-1 Env and inhibition of HIV-1 infectivity was dependent on the cytoplasmic ^222^YxxL^225^ motif of MARCH8 (10). MARCH11 expresses a conserved YxxΦ motif recognized by adaptor protein (AP) μ-subunits and implicated in intracellular protein sorting (32), leading Zhang et al. to propose that the ^222^YxxL^225^ motif may mediate intracellular retention of HIV-1 Env in the TGN, without degradation, after the downregulation of cell surface expression (10). As for HIV-1, anti-IAV activity by MARCH8 required a functional ^222^YxxL^225^ motif but, unlike HIV-1, did not involve the downregulation of viral glycoprotein expression at the cell surface. Using cotransfection approaches, Zhang et al. did not note any major differences in levels of MARCH protein expression between parental and ^222^YxxL^225^ MARCH8 by Western blotting (10); however, in our studies it was clear that inducible MARCH8-^222^YxxL^225^ was expressed at lower levels, although these were still sufficient to downregulate cell surface CD86. Although we cannot uncouple the impact of MARCH protein expression levels from other potential mechanisms of antiviral activity, it is intriguing to speculate that one or more cellular proteins critical for IAV assembly and/or budding might be retained in intracellular compartments after interaction with the ^222^YxxL^225^ motif of MARCH8, thereby modulating the release of infectious virions.

Recently, cotransfection approaches have demonstrated that MARCH8 can impact the expression levels of the IAV HA glycoprotein within mammalian cells. For example, immunoprecipitation studies demonstrated that MARCH8 blocks furin-mediated cleavage of the HA from highly pathogenic avian influenza virus H5N1 (16), whereas seasonal IAV expresses a monobasic cleavage site that is cleaved by trypsin-like enzymes. Moreover, cotransfection of MARCH proteins and IAV HA (strain A/PR/8/1934 (H1N1)) demonstrated that MARCH1, -2, and -8 reduced the levels of HA detected in cell lysates by Western blotting (9). Our studies with MARCH8 and IAV utilized different experimental approaches, relying on DOX-inducible expression of MARCH8 in stably transduced cell lines rather than transient-transfection-based approaches. Moreover, we assessed the impacts of MARCH8 on the expression of multiple virus proteins (in cell lysates, at the cell surface, and in released virions) after infection of cells with IAV. We did not detect reduced levels of HA, NA, M1, or M2 viral proteins in cell lysates or in expression of HA, NA, or M2 at the surface of IAV-infected cells, even though multiple viral proteins were markedly reduced in virions released in the presence of MARCH8. Differences in the timing, expression levels, and/or protein localization of MARCH8 and HA between systems may contribute to these different findings, particularly when considering HA expression after cotransfection compared to its dynamic expression with other virus proteins in the context of IAV-infected cells.

Liu et al. recently reported that MARCH8 inhibits IAV infection by targeting K78 in the viral M2 for ubiquitination and lysosomal degradation (33). While we also report that MARCH8 mediates E3 ligase-dependent inhibition of a late stage in IAV infection, we did not find evidence for modulation of M2 by MARCH8. This might reflect, at least in part, different experimental approaches used in the two studies. First, cotransfection of M2 and MARCH8 or transfection of MARCH8, followed by infection with IAV strain WSN (H1N1), was shown to modulate expression of M2 in cell lysates and at the cell surface (33), whereas inducible MARCH8 did not alter the cell surface M2 on NP^+^ cells following infection with H3N2 (Fig. 6) or with H1N1 (data not shown) viruses. Second, introduction of K78R into A/PR8 or A/WSN viruses abolished restriction in A549 cells with constitutive MARCH8 overexpression, whereas we report that Udorn/72 bearing substitutions in the viral M2 (K49R, K60R, and K78R), alone or simultaneously with K-to-R substitutions in all cytoplasmic K residues of HA, NA, and M2, were all restricted by inducible MARCH8. Finally, while Lui et al. reported virus strains lacking K78 to be resistant to MARCH8 restriction, we show that inducible MARCH8 significantly reduced the infectivity of seasonal H1N1 lacking K78 (A/New Caledonia/1999 and A/Solomon Islands/2006 both have E78). Moreover, inducible MARCH8 also reduced the infectivity of H1N1pdm09 strains (A/Tasmania/2009 and A/Fiji/2016 both have Q78) by >60 and >90%, respectively (data not shown). Given that MARCH8 has also been implicated in modulating HA expression (9) and cleavage (16), it seems likely that the approaches used to overexpress or modulate MARCH8, as well as the particular cell types and virus strains used, may impact the mechanisms underlying MARCH8-mediated inhibition of IAV.

MARCH1 and MARCH8 are generally expressed in different cell types, but are reported to target identical proteins, including CD86 and TfR, among others (1). While MARCH1 and -8 also restrict HIV-1 by similar mechanisms (9, 11, 14), we show that MARCH8, but not MARCH1, mediated potent antiviral activity against IAV. MARCH1 is relatively unstable at mRNA and protein level (22, 34), suggesting that rapid turnover might affect protein expression levels and hence anti-IAV activity. We focused on the N-CT of MARCH1 and -8, given that it is the most variable region and that it has been implicated in the stability and turnover of mouse MARCH1 (22). IAV restriction was lost if the MARCH8 N-CT was deleted, whereas its substitution into a MARCH1 backbone resulted in potent inhibition of IAV. It is important to note that deletion of the MARCH8 N-CT did not have major impacts on MARCH protein expression. In contrast, deletion of MARCH1 N-CT or its substitution into a MARCH8 backbone resulted in increased or reduced levels of MARCH protein expression, respectively. Consistent with findings for mouse MARCH1 (22), expression of the MARCH1 N-CT was clearly associated with reduced levels of MARCH protein expression. Deletion of the MARCH1 N-CT resulted in increased MARCH protein levels and acquisition of anti-IAV activity, suggesting that when its N-CT is removed, additional domains of MARCH1 can restrict IAV. Although difficult to uncouple the impacts of the MARCH1 N-CT on MARCH protein levels versus other mechanisms of anti-IAV activity, all cell lines expressing mutant/chimeric MARCH proteins potently downregulated cell surface CD86. Irrespective of IAV restriction, this important control confirms that different MARCH proteins were expressed at sufficient levels to effect at least one of the known biological functions shared by MARCH1 and -8.

Whereas MARCH8 downregulates envelope glycoproteins from a broad range of viruses, with the exception of VSV-G and MLV p15E, little is currently known regarding the specific protein targets associated with virus restriction. Moreover, when the protein target is known, it is becoming increasingly clear that complex interactions between MARCH protein domains and cellular/viral proteins determine virus restriction. Mouse MARCH1 and -8 both target the MLV p15E envelope glycoprotein; however, MARCH1 interactions occurred independently of its TM domains, whereas MARCH8 interactions occurred via the C-terminal TM domain (TM2) (9). Thus, even when two different MARCH target the same virus glycoprotein, they utilize distinct domains for binding and antiviral activity. Our studies have focused on the N-CT domain of MARCH1 and -8, demonstrating that the MARCH8 N-CT is essential for its anti-IAV activity. We also show that the MARCH1 N-CT modulates MARCH protein levels, and its removal is associated with increased protein expression and the acquisition of IAV restriction. These studies identify a key MARCH protein domain involved in IAV restriction and provide further insights regarding the complexities associated with the molecular mechanisms underpinning MARCH-mediated restriction of different viruses.

## MATERIALS AND METHODS

### Cells.

293T cells (ATCC CRL-3216) were maintained and passaged in Dulbecco modified Eagle medium (Gibco) supplemented with 10% (vol/vol) fetal bovine serum (FBS), 2 mM l-glutamine (Gibco), and 1 mM sodium pyruvate (Gibco). For cells engineered to express DOX-inducible MARCH proteins, Tetracycline System-Approved FBS (TaKaRa Bio USA) was used. Madin-Darby canine kidney (MDCK) cells (ATCC CCL-34) were maintained and passaged in RPMI 1640 medium supplemented with 10% (vol/vol) FBS (Gibco), 2 mM l-glutamine, and 1 mM sodium pyruvate.

### Viruses.

Seasonal IAV strains were used in this study: H3N2 (A/Udorn/307/72, A/Port Chalmers/1/73, A/Beijing/353/89, A/Guangdong/25/93 and A/New York/55/04) and H1N1 (A/Brazil/11/78, A/New Caledonia/20/99, and A/Solomon Islands/3/06). Viruses were obtained from the WHO Collaborating Centre for Reference and Research on Influenza (WHO CCRRI), Melbourne, Australia. All viruses were grown in the allantoic cavity of 10- to 11-day-embryonated hen’s eggs by standard procedures, and viral titers were determined by a plaque assay in MDCK cells (35); the results are expressed as PFU/ml. To generate recombinant viruses by reverse genetics, we used pHW2000 plasmids containing the eight genes of A/Udorn/307/1972 (H3N2). All plasmids were kindly provided by Robert Webster (St. Jude Children’s Research Hospital, Memphis, TN). Site-directed mutagenesis (SDM) was used to introduce appropriate mutations into genes to allow for lysine-to-arginine (K-to-R) substitutions in the viral HA, NA, M1, and M2 proteins. To create HA lacking its CT (HA ΔCT), a stop codon was introduced after the last residue of the transmembrane domain. To remove the CT of NA (NA ΔCT), specific primers were used to delete the five N-terminal residues after the initiation codon methionine. Recombinant viruses were generated by standard procedures (36), and final virus stocks were generated in embryonated eggs. Genes of interest were sequenced to confirm the incorporation of the relevant mutation(s). The CAN97-83 hMPV strain was propagated in LLC-MK2 cells, and titers of infectious virus were determined by titration on LLC-MK2 monolayers, followed by immunofluorescence staining with a monoclonal antibody (MAb) against the hMPV N protein (MAB80121; Merck Millipore, MA), as described previously (37).

### Generation of cell lines with DOX-inducible protein expression.

A three-step cloning strategy was required to generate appropriate lentiviruses (38). First, coding sequences of MARCH1 isoform 2 (CCDS3806.1) and MARCH8 (CCDS7213.1) containing an N-terminal FLAG tag were synthesized as “geneblocks” (GeneArt Strings DNA Fragments; Invitrogen) and cloned into pcDNA3.1 (Invitrogen). Each coding sequence was then amplified by PCR to introduce a HpaI restriction site and then cloned into pTRE-tight, which carries a tight TRE promoter containing seven tet operator sequences that confer DOX inducibility. Fragments containing the TRE promoter and gene of interest were then cloned into the pFUV1mCherry lentivirus transfer plasmid, which also expresses mCherry under the control of the constitutive ubiquitin promoter. Plasmids were kindly provided by Marco Herold (Walter and Eliza Hall Institute of Medical Research, Melbourne, Australia). A similar strategy was used to generate stable control (CTRL) cells with DOX-inducible expression of cytoplasmic hen egg ovalbumin lacking the sequence for cell surface trafficking (39). Lentiviral particles were produced by cotransfecting the packaging plasmids pMDL, pRSV-REV, and pMD2.G, along with the appropriate pFUV1mCherry transfer plasmid using Lipofectamine 2000 (Invitrogen), according to the manufacturer’s instructions. At 48 h posttransfection, the cell supernatants harvested were used to transduced 293T cells, and mCherry-positive cells were sorted 72 h later using a BD FACSAria III cell sorter (BD Biosciences) and expanded for use.

### Detection of DOX-inducible MARCH proteins by flow cytometry.

DOX-inducible 293T cells were seeded in 24-well tissue culture plates (Nunc) and cultured overnight. To induce protein expression, cells were incubated in media containing 1 μg/ml of DOX (Sigma-Aldrich) for 24 h at 37°C. In some experiments, a 10 μM concentration of the proteasomal inhibitor MG132 (Sigma-Aldrich) was also added 20 h after DOX induction to reduce intracellular protein degradation. At 24 h after DOX induction, the cells were detached and stained with the fixable viability dye eFluor 780 (eBioscience), washed, and fixed with 2% (vol/vol) paraformaldehyde in PBS. After permeabilization in 0.5% (vol/vol) Triton X-100, the cells were stained with anti-FLAG-allophycocyanin (APC) MAb (clone L5; BioLegend), washed, and analyzed by flow cytometry. Samples were acquired on a LSRFortessa flow cytometer (BD Bioscience) and analyzed using FlowJo analysis software version 10.6.2.

### Detection of MARCH protein expression by immunoprecipitation and Western blotting.

Cells cultured for 24 h in media (No DOX) or supplemented with 1 μg/ml DOX (DOX) or with DOX and MG132 for the last 4 h (DOX+MG132) were lysed with ice-cold lysis buffer (50 mM Tris-HCl, 1% [vol/vol] Triton X-100, 150 mM NaCl, 1 mM CaCl_2_, 1 mM MgCl_2_, 10% glycerol) supplemented with protease inhibitors (cOmplete Mini Protease Inhibitor Cocktail; Roche) for 20 min on ice and centrifuged to remove cell debris. For immunoprecipitation, protein G-Sepharose beads (GE Healthcare) were incubated with anti-FLAG MAb (clone M2; Sigma-Aldrich) for 2 h at 4°C and washed three times to remove unbound MAb. Anti-FLAG-coupled Sepharose beads were then incubated with clarified supernatants overnight at 4°C and washed extensively with lysis buffer prior protein elution. To elute the bound proteins, the beads were incubated with 2× reducing sample buffer (100 mM Tris, 4% [vol/vol] sodium dodecyl sulfate [SDS], 0.1% [wt/vol] bromophenol blue, 20% [vol/vol] glycerol and 200 mM dithiothreitol) for 10 min at 95°C, and the eluted proteins were then subjected to SDS-PAGE using a 12.5% (vol/vol) acrylamide/bis-acrylamide gel, followed by transfer to polyvinylidene difluoride membranes (Immobilon-P; Merck Millipore). Membranes were blocked for 1 h and incubated overnight at 4°C with anti-FLAG MAb-conjugated to horseradish peroxidase (HRP; clone M2; Sigma-Aldrich). Bound antibodies were detected by chemiluminescence, using the SuperSignal West Pico chemiluminescent substrate (Thermo Fisher Scientific) according to the manufacturer’s instructions. Images were acquired using an Amersham Imager 600 (GE Healthcare).

### Modulation of cell surface CD86 expression.

To evaluate the functionality of MARCH proteins after DOX induction, flow cytometry was used to monitor cell surface expression of CD86. DOX-inducible 293T cells were transiently transfected with pHAGE-CD86-ZsGreen (kindly provided by Melissa Call, Walter and Eliza Hall Institute of Medical Research, Melbourne, Australia) using Lipofectamine 2000 according to the manufacturer’s instructions. pHAGE-CD86-ZsGreen contains an internal ribosome entry site (IRES) allowing for simultaneous expression of CD86 and the fluorescent protein ZsGreen. At 12 h posttransfection, the cells were incubated in media (No DOX) or DOX induced. At 48 h posttransfection, the cells were stained with fixable viability dye eFluor 780 and anti-CD86 conjugated to Brilliant Violet 711 MAb (clone 2331 [FUN-1]; BD Biosciences) and analyzed by flow cytometry.

### Virus infection assays.

Cells seeded in 24-well tissue culture plates were washed and then inoculated with IAV at the indicated multiplicity of infection (MOI) in PFU/cell. After incubation for 1 h at 37°C, the cells were washed to remove residual inoculum and incubated in serum-free media. In some experiments, the medium was supplemented with 0.5 μg/ml TPCK (tolylsulfonyl phenylalanyl chloromethyl ketone) trypsin (Worthington Biochemical) to promote multicycle replication. To evaluate the early stages of virus replication, the percentage of cells expressing newly synthesized IAV nucleoprotein (NP) was measured by flow cytometry at 8 hpi. Virus-infected cells were washed, detached, and stained with fixable viability dye eFluor 780 (eBioscience) and then fixed, permeabilized, and stained with anti-IAV NP conjugated to FITC (clone 431; Abcam). Samples were analyzed by flow cytometry. To evaluate late stages of IAV replication, cell supernatants were collected at the indicated times and clarified by centrifugation, and titers of the infectious virus in cell-free supernatants were determined by a standard plaque assay (35) or by a ViroSpot (VS) assay as described previously (40). The VS assay was also adapted to determine titers of hMPV. Briefly, MDCK (for IAV) or Hep2 cells (for hMPV) in 96-well tissue culture plates were incubated with 10-fold dilutions of samples containing virus and then incubated for 24 h at 37°C under a carboxymethylcellulose overlay. After fixation in cold 80% (vol/vol) acetone, the plates were stained with MAbs specific for the NP (IAV) or N (hMPV), followed by HRP-conjugated rabbit anti-mouse antibody (Dako, Denmark). Virus-infected cells were visualized by incubating plates with KPL TrueBlue peroxidase substrate (Life Sciences, Inc., Milford, MA) and scanned with a CTL ImmunoSpot analyzer (CTL, Shaker Heights, OH). Spots (10 to 150/well) were counted manually using an ImageJ cell counter, and virus titers are expressed as VS/ml.

### Quantification of vRNA by RT-qPCR.

To determine the number of copies of vRNA in the cell supernatant, vRNA was extracted from clarified cell culture supernatants using the QIAamp Viral RNA minikit (Qiagen) according to the manufacturer’s instructions. To calculate the number of copies of the segment coding for the matrix (M) or the hemagglutinin (HA) proteins, 4-μl portions of vRNA were amplified using specific primers and probes in combination with a SensiFAST Probe Lo-ROX One-Step kit (Bioline) according to the manufacturer’s instructions. The qPCR was performed using the QuantStudio 7 Flex real-time PCR system (Applied Biosystems). Copy numbers of different vRNAs genes were calculated by generating a standard curve using 10-fold serial dilutions of plasmid containing DNA encoding the IAV M gene from A/California/7/2009 (H1N1pdm09) or the IAV HA gene from A/Beijing/353/89. To determine infectivity per particle, the ratio between the titer of infectious virus and the number of copies of the M and/or HA segment was calculated, as described previously by Bedi et al. (15).

### IAV infection via plasma membrane acid bypass.

DOX-inducible 293T cells were seeded in poly-l-lysine-treated tissue culture plates and cultured for 24 h in media (No DOX) or DOX induced (DOX). The cells were washed and incubated with serum-free medium supplemented with 10 mM NH_4_Cl for 15 min at 4°C and then washed and infected with IAV (MOI of 25 PFU/cell) for 1 h at 4°C. The cells were next washed extensively before incubation with ice-cold serum-free media (pH 7.4) or fusion buffer (20 mM HEPES, 2 mM CaCl_2_, 150 mM NaCl, 20 mM citric acid monohydrate-sodium citrate tribasic dehydrate [pH 5.0]) for 10 min at 37°C to induce virus fusion at the plasma membrane. After low-pH treatment, the cells were washed and incubated in medium containing 10 mM NH_4_Cl to prevent subsequent endosomal virus entry and fusion. Cells collected at 8 hpi were analyzed for IAV infection by flow cytometry, while infected cell supernatants were harvested at 24 hpi and titrated by plaque assay.

### Generation of MARCH8 mutants and chimeric MARCH1 and -8 proteins.

Two MARCH8 RING-CH mutants were generated: MARCH8-CS (20) and MARCH8-W114A (21). For MARCH8-CS, geneblocks were synthesized wherein the codon TGT was replaced by the codon AGC to replace cysteine (C) residues at positions 80, 83, 97, 99, 110, 123, and 126 with serine (S) residues. For MARCH8-W114A, tryptophan (W) at position 114 was replace by alanine (A) by SDM using the pcDNA3.1-MARCH8 plasmid and degenerate primers (forward, 5′-GCCTGTCTGCAGCAGGCGATCAAGAGCAGCGACAC-3′; reverse, 5′-GTGTCGCTGCTCTTGATCGCCTGCTGCAGACAGGC-3′) carrying the W114A mutation (underlined). In addition, two tyrosine motif mutants (MARCH8_-2_^22^AxxL^225^ and MARCH8-^232^AxxV^235^) were also generated by SDM. MARCH8-^222^AxxL^225^ was generated using degenerate primers (forward, 5′-GTGCAGTGCAAAGTGGCCGTGCAGCTGTGGAAGC-3′; reverse, 5′-GCTTCCACAGCTGCACGGCCACTTTGCACTGCAC-3′) and for MARCH8-^232^AxxV^235^ (forward, 5′-GCGGCTGAAGGCCGCCAACAGAGTGATCTAC-3′; reverse, 5′-GTAGATCACTCTGTTGGCGGCCTTCAGCCGC-3′). SDM was performed using the *Pfu* DNA polymerase (Agilent Technologies) according to the manufacturer’s instructions. PCR products were then treated with DpnI (NEB) to digest template plasmid DNA that did not carry the mutation required, and mutagenesis was confirmed by Sanger sequencing (Australian Genome Research Facility [AGRF]). For N-CT domain mutants, the N-CT domains of MARCH1 and MARCH8 were identified based on the position of their respective RING-CH domains. To generate MARCH1_ΔN-CT and MARCH8_ΔN-CT, PCR was used to amplify MARCH1 and MARCH8, which lacked the N-CT (forward primers 5′-AAGGGATCCCCCAGCACCCAGGACATCTGCAG-3′ and 5′-AAGGGATCCCCCAGCAGCCAGGACATCTGCCGG-3′, respectively; reverse primer specific for pcDNA3.1). Chimeric mutants MARCH1_M8 N-CT and MARCH8_M1 N-CT were synthesized as geneblocks. Cell lines with DOX-inducible expression of mutant versions of MARCH8 were generated as described above.

### Detection of viral proteins in supernatants and cell lysates of IAV-infected cells by Western blotting.

Cells cultured for 24 h in media (No DOX) or supplemented with 1 μg/ml DOX (DOX) were washed and infected with IAV (MOI of 5 PFU/cell), and the supernatants harvested at 24 hpi were clarified by centrifugation (3,000 rpm, 15 min, 4°C) and then concentrated by ultracentrifugation through a 30% (vol/vol) cushion sucrose (120,000 × *g*, 2 h at 4°C, SW32 Ti rotor, Optima XPN-100 ultracentrifuge [Beckman Coulter]) and resuspended in PBS. IAV-infected cells were lysed as described above. Cell lysate or concentrated viral particles were mixed with reducing sample buffer and subjected to SDS-PAGE and Western blotting with goat anti-HA (NR-3118; BEI Resources), goat anti-NA (NR-3137, BEI resources), mouse anti-NP (clone A1 and A3; Bio-Rad), mouse anti-M1 (clone GA2B, Bio-Rad) or mouse anti-M2 (clone 14C2, Abcam), followed by anti-mouse IgG-HRP (Agilent Dako) or anti-goat IgG-HRP (Abcam). Bound antibodies were detected by chemiluminescence, and densitometry was performed using ImageJ software.

### Hemagglutination and NA enzymatic assays.

Hemagglutination assays were performed in round-bottom 96-well plates using 1% (vol/vol) turkey erythrocytes in PBS by standard procedures. The results are expressed as hemagglutination units (HAU). NA activity was determined using a fluorometry-based assay that measures the amount of fluorescent 4-MU cleaved after incubation with MUNANA substrate (41). Fluorescence measurements were acquired using a Fluoroskan Ascent fluorometer (Thermo Fisher Scientific), and NA activity was recorded as relative fluorescence units (RFU).

### Cell surface expression of IAV glycoproteins in infected cells by determined flow cytometry.

Cells cultured for 24 h in media (No DOX) or supplemented with 1 μg/ml DOX (DOX) were washed and infected with IAV (MOI of 1 PFU/cell). At 8 or 24 hpi, the cells were washed, detached, and stained with an MAb against IAV-M2 (clone 14C2;, Abcam), a biotinylated MAb against IAV HA (clone C1/1), or a goat polyclonal antiserum (pAbs) against IAV NA (NR-3137; BEI Resources) in combination with fixable viability dye eFluor 780 for 30 min on ice. Cells were washed and incubated with either anti-mouse IgG-phycoerythrin (BD Bioscience) to detect viral M2, V450-conjugated streptavidin (BD Bioscience) to detect viral HA, or anti-goat IgG Alexa Fluor 647 (Invitrogen) to detect viral NA. Cells were then washed, fixed with 2% (vol/vol) paraformaldehyde, permeabilized with 0.5% (vol/vol) Triton X-100, stained with anti-IAV NP fluorescein (FITC) MAb (clone 431; Abcam), and analyzed by flow cytometry.

### Detection of endogenous *MARCH8* mRNA expression by qRT-PCR.

To generate hMDMs, peripheral blood mononuclear cells were isolated from healthy blood donors using Ficoll-Paque density gradient centrifugation, followed by positive selection of CD14^+^ monocytes using CD14 microbeads (Miltenyi Biotec). To obtain classically activated hMDMs, CD14^+^ monocytes were cultured in RPMI supplemented with 10% human serum (Sigma) for 6 to 7 days, with 50 ng/ml IFN-γ and 10 ng/ml lipopolysaccharide added after 4 and 5 days, respectively. 293T cells or hMDMs were treated with 500 U/ml IFN-α (Lonza), infected with IAV (Beij/89 [MOI 10 PFU/cell]), or incubated with medium only (mock). After 2 or 24 h, the total RNA was extracted using a RNeasy minikit (Qiagen) and converted to cDNA using a SensiFAST cDNA synthesis kit (Bioline). SYBR green-based qPCR was used to analyze the expression of *MARCH8* and *IFITM3* relative to three housekeeping genes—*GAPDH* (glyceraldehyde 3-phosphate dehydrogenase), *RPL13A* (ribosomal protein L13a), and *TBP* (TATA-binding protein)—using a SensiFAST SYBR Lo-ROX kit. The specific primers used were as follows: *MARCH8*, forward (5′-AGCCACTGAGAAAATGGGAGAAG-3′) and reverse (5′-TGTCACTGAGCACATGATCTTCC-3′); *IFITM3*, forward (5′-ATCGTCATCCCAGTGCTGAT-3′) and reverse (5′-ACGTGGGATACAGGTCATGG-3′); *GAPDH*, forward (5′-TGAAGGTCGGAGTCAACGG-3′) and reverse (5′-GGCAACAATATCCACTTTACCAGAG-3′); *RPL13a*, forward (5′-GCCCCTGTTTCAAGGGATAA-3′) and reverse 5′-CCTCGACCATCAAGCACCA-3′; and *TBP*, forward (5′-GCACTGATTTTCAGTTCTGG-3′) and reverse (5′-GCTGGAAAACCCAACTTCTGT-3′). Expression of inducible *MARCH8* was determined using the primers ind_MARCH8 forward (5′-GACGATGACAAGGGATCCATGAG-3′) and ind_MARCH8 reverse (5′-GCTTCTGTACACTCTGGCGG-3′). Data acquisition was performed using the QuantStudio 7 Flex real-time PCR system (Applied Biosystems).

### siRNA knockdown of endogenous *MARCH8*.

hMDMs and 293T cells were transfected with 1 μM siRNA specific for MARCH8 or nontargeting control (NTC; Accell SMART pool; Dharmacon, Lafayette, CO) using Lipofectamine RNAiMAX (Thermo Fisher Scientific), according to the manufacturer’s instructions. hMDMs were treated at day 3 after seeding. At 72 h post-siRNA, the cells were infected with IAV Beij/89 (hMDMs [MOI 2.5], 293T cells [MOI 0.01]) and cultured in the presence of 0.5 μg/ml TPCK trypsin. At 2, 24, and 48 hpi, cell lysates (qPCR) and supernatants (plaque assay) were harvested for analysis.

### Statistical analysis.

Graphs and statistical analysis (as indicated in the figure legends) were performed using Prism version 9.0.2 (GraphPad Software).
